# Surgical outcomes and complications associated with ovariectomy in the southern stingray *Hypanus americanus*

**DOI:** 10.3389/fvets.2024.1410421

**Published:** 2024-07-22

**Authors:** Robert H. George, Chris Buckner, Katherine Baine, James Steeil, Stacia White, Tim Handsel, Jennifer T. Wyffels

**Affiliations:** ^1^Ripley’s Aquarium of Myrtle Beach, Myrtle Beach, SC, United States; ^2^Ripley’s Aquarium of the Smokies, Gatlinburg, TN, United States; ^3^Smithsonian’s National Zoo and Conservation Biology Institute, Washington, DC, United States; ^4^Ripley’s Aquariums, Orlando, FL, United States

**Keywords:** ovary, cyst, histotroph, dystocia, reproductive disease, contraception

## Abstract

Southern stingrays (*Hypanus americanus*) are relatively large rays that are common and popular in public aquariums because of their size and gentle nature. In aquariums, as well as in the wild, female southern stingrays are fecund. They have a short gestation cycle and can sustain multiple pregnancies each year, each culminating with 2–10 young. This reproductive rate could quickly outpace capacity in managed care and result in a ray surplus. To prevent overpopulation, many aquaria have resorted to single sex groups with a preference for female-only populations. This is an effective way to control population growth, but forces the maintenance of two separated populations of rays; for females this interrupts normal reproductive cycling and replaces it with a protracted non-pregnant condition. An additional consideration is development of reproductive disease in females which is recognized by an enlarged, misshapen, and congested ovary with an abundance of cystic structures and an enlarged uterus with a thickened wall that is often filled with histotroph despite a non-pregnant status. There are no effective long-lasting medical treatments for this type of reproductive disease and mortality is often the result. This report describes a surgical technique for ovariectomy in southern stingrays including outcomes and complications. Ovariectomy as a surgical method prevents unwanted reproduction and has the benefit of reducing reproductive pathologies commonly observed in southern stingrays as they age. Seven stingrays 1–5.2 years old and 42–83.5 cm disc width underwent ovariectomy. After anesthesia, the ovary and a small amount of epigonal was excised via a left para-lumbar incision. Four of the seven rays survived five or more years post-procedure. Two rays died acutely of coelomitis and one ray died of complications unrelated to the procedure. This report details a surgical procedure for ovariectomy in southern stingrays including outcomes, complications, and recommendations.

## Introduction

Southern stingrays (*Hypanus americanus*) are relatively large rays that are common and popular in public aquariums because of their size and gentle nature. They are often maintained in shallow exhibits that offer guests a chance to touch and interact with them through guest interaction programs. In aquariums, as well as in the wild, female southern stingrays are long-lived (1) and fecund (2). Females have a functional left ovary and uterus, rudimentary and likely non-functional right ovary, and vestigial right uterus (3–5). Southern stingrays in managed care have a relatively short gestation cycle and can sustain multiple pregnancies in any given year, each culminating with 2–10 young (2). The average gestation calculated from the interval between successive parturition dates for seven females at Ripley’s Aquarium of Myrtle Beach is 140 ± 9 days (Table 1). The number of young scales with female size so as rays grow their fecundity effectively increases (2, 3). This reproductive rate could quickly outpace capacity in managed care and result in a ray surplus. To prevent overpopulation, many aquariums have resorted to single sex exhibits with a preference for female-only populations. This can be attributed to the larger size of females compared to males (1) but also because of the potential for injuries from conspecific male sexual aggression.

**Table 1:** Parturition dates and gestation time for southern stingrays at Ripley’s Aquarium of Myrtle Beach.

Ray ID	Successive parturition dates				Total days	Average gestation ± SD
	1	2	3	4		
091*602*093	05/25/08	10/18/08			146	146
088*262*550	04/03/08	08/18/08			137	137
091*612*103	01/06/08	05/24/08	09/25/08		263	132 ± 11
055*823*843	06/05/08	10/02/08	02/12/09		252	126 ± 10
056*572*292	12/20/07	05/15/08	09/25/08	02/21/09	429	143 ± 9
056*802*312	12/15/07	05/07/08	10/29/08	03/23/09	464	155 ± 18
056*101*838	01/24/08	05/28/08	10/08/08	03/22/09	423	141 ± 21

Southern stingrays in the wild reproduce asynchronously with ovulation and parturition observed throughout the year (3). However, for southern stingrays in a single sex population, there is a protracted period of non-pregnancy that may contribute to the development of reproductive disease (5). During this non-pregnant period, the left ovary continues to mature follicles. Continuous follicle production without a corresponding rate of ovulation results in the accumulation of follicles and ovarian mass over time (personal observation). The ovary often becomes cystic and can achieve huge dimensions, weighing up to a kilogram (5). Ovarian tissues are thin and friable, and when ovaries attain such a large size, they are prone to tearing which can lead to catastrophic hemorrhage and death.

Reproductive disease also affects the uterus which often continues to produce histotroph meant to supplement the nutrition of developing embryos. Ultrasonography often reveals a large hypoechoic histotroph-filled uterus (5). The vestigial right uterus may also be similarly affected. Since the ray’s coelom may become distended ventrally as well as dorsally from the swollen uteri, rays sometimes develop pressure sores when resting on the exhibit floor. For several southern stingrays, the pressure sores became severe and weakened the coelomic wall which ruptured. The loss of coelomic integrity was followed by the development of severe coelomitis that was nonresponsive to antibiotic therapy and resulted in death (personal observation). The accumulation of histotroph may be symptomatically treated by cannulating the cervix and evacuating the uterus but the fluid accumulation often recurs after only a few months (personal observation). There are no known chemical contraceptive methods effective for elasmobranchs and therefore surgical methods were explored (6). As an alternative to pharmacological contraception, ovariectomy controls unwanted reproduction and has the benefit of reducing reproductive pathologies commonly observed in southern stingrays as they age. To manage the population size and prevent development of reproductive disease, ovariectomy was pursued.

## Materials and methods

### Animals

Southern stingrays (*n* = 7) were considered candidates for ovariectomy if they were less than 85 cm disc width (DW), not pregnant, and did not have evidence of reproductive disease.

### Surgical procedure

**Figure 1:**
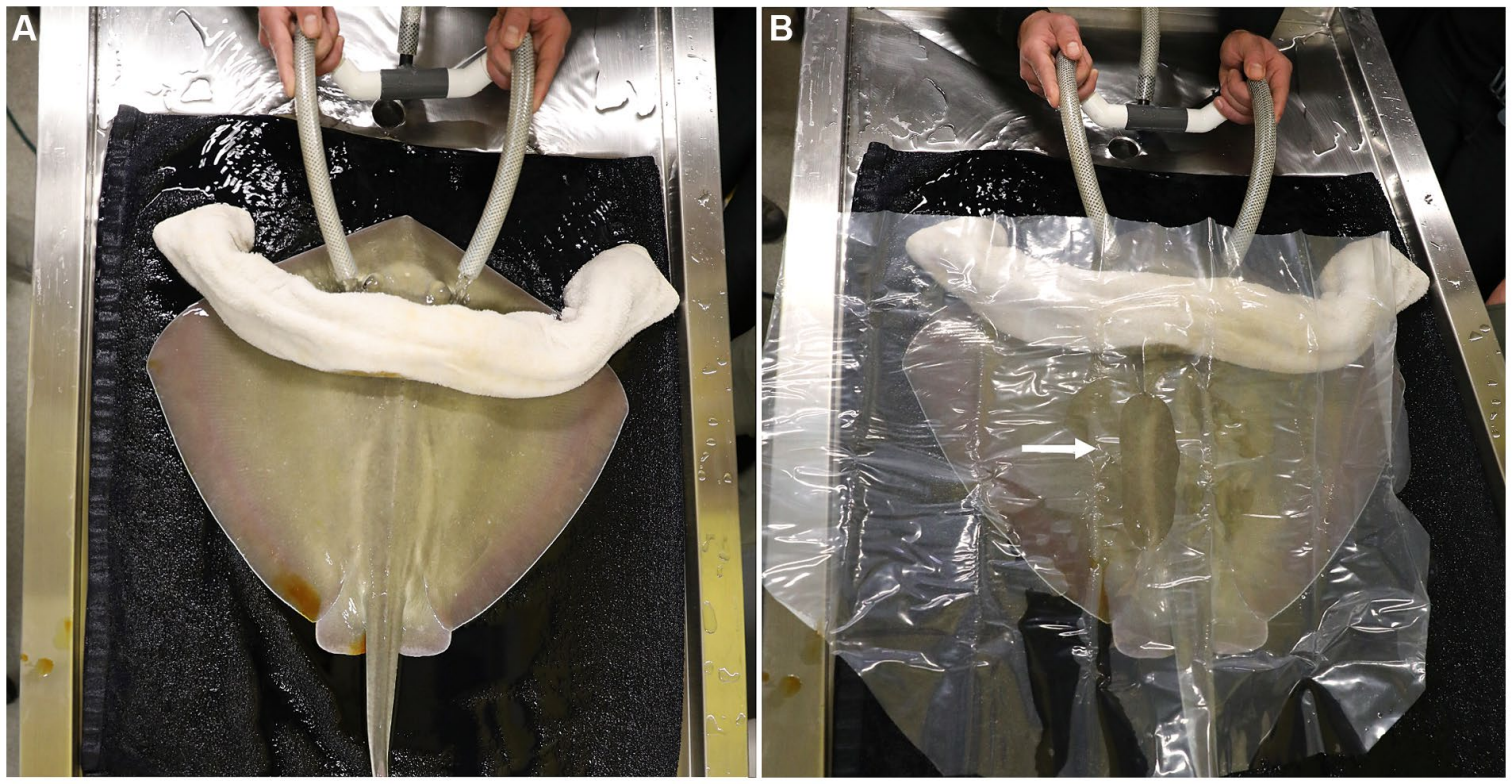
**(A)** The anesthetized southern stingray is placed on a prewetted towel on the surgical surface of the portable medical cart. Seawater was pumped from the anesthetic tank through a ½ inch polypropylene hose to a T adapter from which 1/2 inch hoses are placed into the ray’s spiracles. A rolled and pre-wetted cotton towel was laid caudal to the spiracles to block potential overflow anesthetic water from contaminating the surgical site. **(B)** A clear plastic, sterile drape was sutured in position and the surgical area exposed (arrow) for a 6–8 cm incision initiated just caudal to where the fin rays (ceratotrichia) join with the epaxial muscles parallel to the spinal column and 2–4 cm lateral to the dorsal lumbar muscles.

Candidate rays were captured and placed in a holding tank containing oxygenated seawater from their exhibit. A second tank with oxygenated seawater from the same source was treated with tricaine methanesulphonate (MS-222) (70 ppm; Syndel, Ferndale, Washington, United States) buffered 4:1 with sodium bicarbonate. Once the MS-222 and bicarbonate had dissolved, a ray was added to the anesthetic solution. Anesthesia was deemed sufficient when the ray was minimally responsive to touch while still maintaining spiracular respiration. The ray was lifted out of the anesthetic tank and placed on a pre-moistened towel covering the surface of a custom and portable stingray medical treatment cart adjacent to the holding tanks. A submersible pump, placed in the anesthetic tank pumped seawater through a ½ inch polypropylene hose to a T adapter from which ½ inch hoses were placed into the ray’s spiracles (Figure 1A). Anesthetic seawater flowed through the spiracles, cascaded over the gills and drained from the ray’s gill slits as well as its mouth. The anesthetic seawater drained from the top tray of the medical cart into a catching basin on the bottom tray of the cart and was recycled back into the anesthetic holding tank.

**Figure 2:**
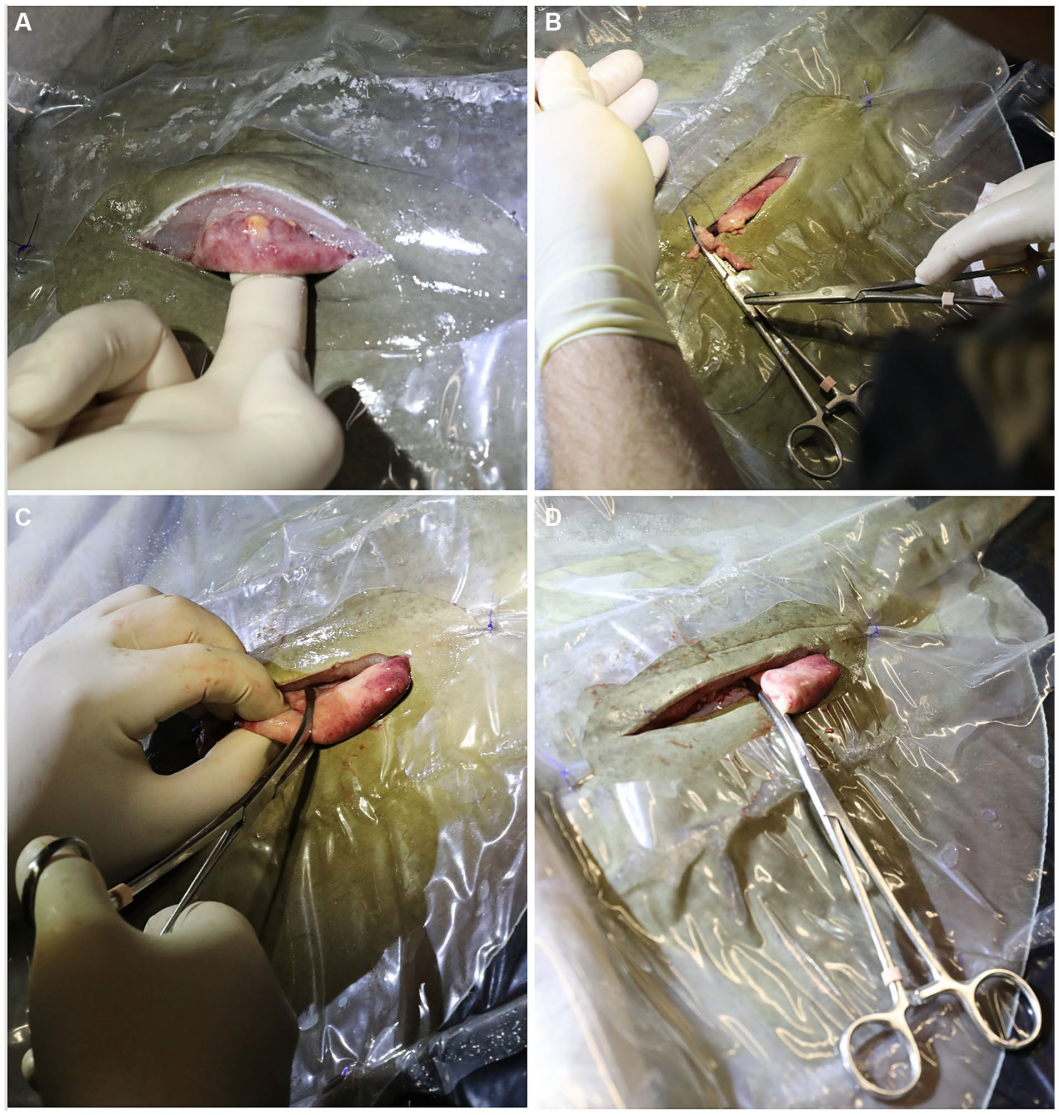
**(A)** After incising the muscle and the peritoneum, the cranial portion of the ovary and the oviduct were dissected free from the dorsal suspensory ovarian ligament. **(B)** Two 6 inch curved Kelly hemostat clamps were placed around the freed tissue anterior to the ovary. Using 3-0 polydioxanone suture (PDS) material, a single encircling ligature was placed anterior to the two clamps and the tissue between them was incised. A second tie was then placed around the clamped blood supply. **(C)** A clamp was placed across the epigonal organ caudal to the junction, an encircling suture was placed around the epigonal organ. **(D)** The ovarian tissue was transected and removed along with a small amount of epigonal tissue.

A rolled and pre-moistened cotton towel was laid caudal to the spiracles to block potential overflow of anesthetic seawater from contaminating the surgical site. The left para-lumbar area was sanitized using gauze soaked in povidone iodine surgical prep solution and applied using light pressure starting at the incision site and circling outward. The povidone application was repeated twice more and a clear plastic, sterile, surgical drape was sutured in position to isolate the surgical area (Figure 1B). A 6–8 cm incision was made using a number 10 blade parallel to the spinal column and 2–4 cm lateral to the dorsal lumbar muscles. The skin incision was initiated just caudal to where the fin rays (ceratotrichia) join with the epaxial muscles. Even though the incision is parallel to the spine, it is unavoidable to incise nerves that run medio-laterally all along the incision site. This does not impair normal swimming patterns post-surgery. After incising the muscle and the peritoneum, the cranial portion of the ovary and the oviduct were dissected free from the dorsal suspensory ovarian ligament (Figure 2A). Two 6-inch curved Kelly hemostat clamps were placed around the freed tissue anterior to the ovary. Using 3-0 polydioxanone suture (PDS) material, a single encircling ligature was placed anterior to the two clamps (Figure 2B) and the tissue between them was incised. A second tie was then placed around the clamped blood supply. The thick anterior pedicle appeared to supply most of the blood to the ovary. A thin suspensory ovarian ligament ran along the ray’s dorsal midline between the ovary and the body wall. It was transected with scissors or blunt dissection and was relatively avascular. Mosquito forceps were applied for hemostasis as necessary. The caudal pole of the ovary was broadly attached to the cranial end of the epigonal organ. A clamp was placed across the epigonal organ caudal to the junction (Figure 2C), an encircling suture was placed around the epigonal organ, and the ovarian tissue was transected and removed along with a small amount of epigonal tissue (Figure 2D).

**Figure 3:**
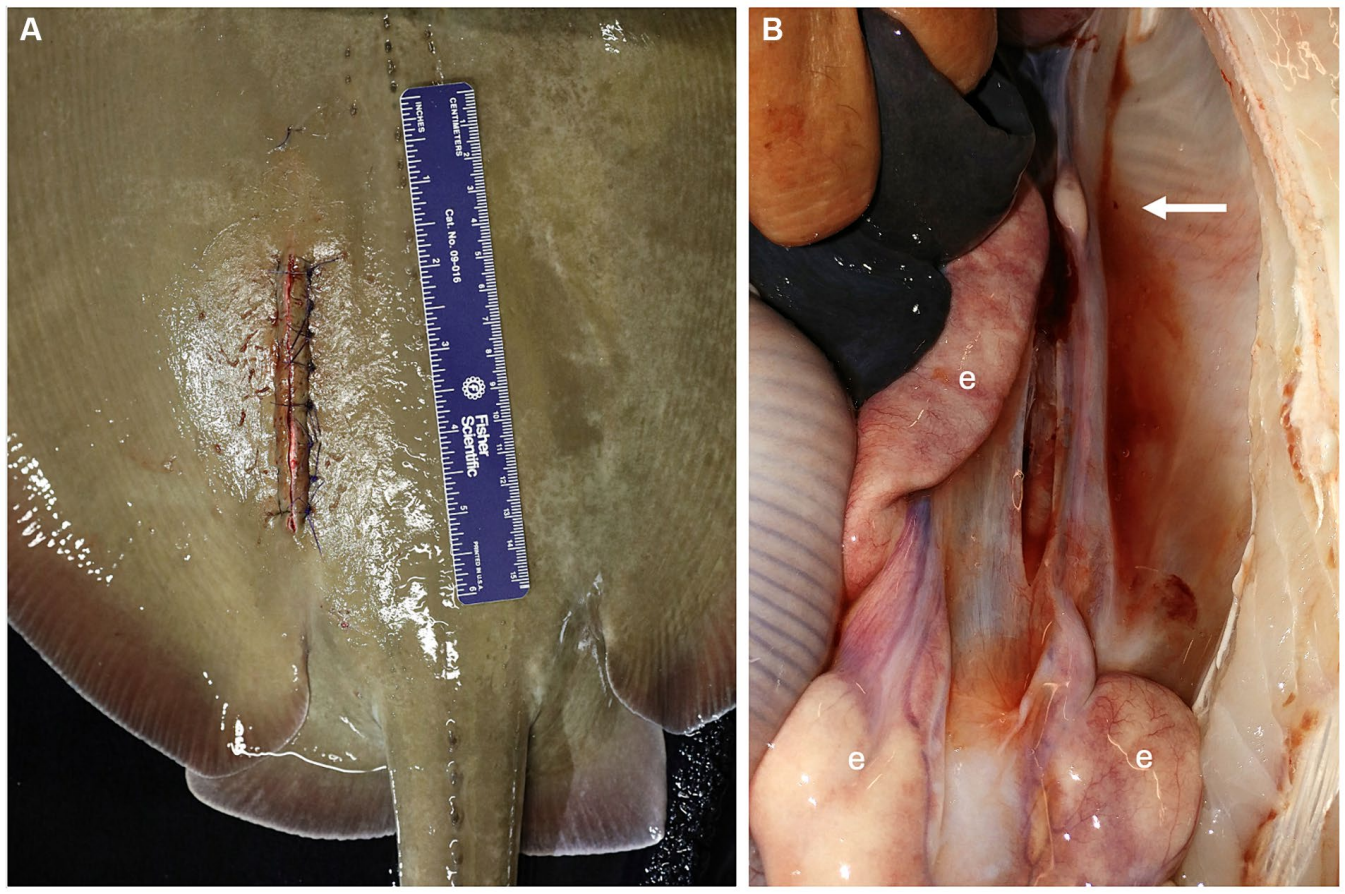
**(A)** The peritoneum was sutured with 3-0 PDS material on a half round taper needle in a simple interrupted pattern and the skin was sutured with 3-0 PDS material on a cutting needle in a simple interrupted pattern. **(B)** Lack of ovarian tissue was confirmed during necropsy 5.1 years after surgical ovariectomy. The right epigonal organ (e) extends cranial to caudal in the coelom but is reduced on the left side where the ovary was removed (arrow).

The peritoneal membrane was sutured with 3-0 PDS material on a half round taper needle in a simple interrupted pattern. The muscular layers were not directly sutured as the tissues were too weak to hold sutures. At this time, 1–2 mL of 2% lidocaine were dripped into the incision for post-surgical analgesia. The skin was sutured with 3-0 PDS material on a cutting needle in a simple interrupted pattern (Figure 3A). It was important to get good tissue apposition to prevent saltwater intrusion so care was taken to place the sutures relatively close together and tighter than would be done for mammals. Intramuscular injection of NSAIDs (ketoprofen 2 mg/kg or meloxicam 2 mg/kg) and antibiotics (ceftiofur 8 mg/kg) were administered, the spiracular hoses removed, and the ray moved to the adjacent holding or recovery tank. Once the ray responded to touch by swimming away in a normal manner it was returned to the original exhibit. The surgery site did not require post-operative care and sutures were removed after 1 month.

## Results

During the development of this surgical procedure, 3 rays (54–65 cm DW, 6.5–8.8 kg, 3–3.9 years old) underwent successful exploratory laparotomies with the intent to evaluate the detailed anatomy of the ovary and associated epigonal tissue and assess the practicality of an ovariectomy but were not ovariectomized, and 7 rays were subsequently ovariectomized (Table 2). Average surgery time was 30 ± 11 min (*n* = 6, average ± standard deviation) and ranged from 17–49 min. Of the ovariectomized rays, one died 22 days post procedure from coelomitis, one died 21 days post-procedure from coelomitis, and a third in poor body condition at the time of surgery died 21 days post-procedure of unrelated health complications. Four rays survived long-term after surgery, two surviving 5.1 and 6.0 years, and two were alive at the time of writing, 5.2 and 10.9 years post-procedure. Among this relatively small cohort of ovariectomized southern stingrays, none became pregnant, and no evidence of regeneration of functional ovarian tissue or rudimentary and likely non-functional vestigial ovarian tissue has been observed at necropsy (Figure 3B) or ultrasonographically during annual wellness exams.

**Table 2:** Southern stingray age and size at ovariectomy with survivorship post-ovariectomy ordered by ray disc width and body weight.

Ray ID	Age (years)	Disc width (cm)	Body weight (kg)	Survivorship post-ovariectomy
MB-DA-18-13-F	1	42	2.1	Alive, 5.2 years
MB-DA-10-03-F	4.6	53	4.3	22 days
MB-DA-10-10-F	4.5	54	4.7	21 days
MB-DA-08-28-F	4.8	54	7.8	Alive, 10.9 years
MB-DA-09-19-F	4.2	64	8	5.1 years
MB-DA-07-03-F	5.2	70.3	10.73	21 days
GB-DA-08-05-F	4.9	83.5	20.5	6.0 years

## Discussion

Ovariectomy of southern stingrays is a practical technique that can be employed to prevent unwanted reproduction and has the added benefit of reducing reproductive pathologies commonly observed in southern stingrays as they age. Mature stingrays with a well-developed ovary make it challenging to safely complete the surgery because of the large ovarian mass and corresponding blood supply compared to immature or maturing rays. The smallest and most immature ray also complicated the surgery because of the difficulty to identify the margins of the ovary from the epigonal organ. To help choose the ideal surgical candidate, ovary and ovarian follicle size may be evaluated pre-operatively by ultrasonography for rays nearing maturity based on disc width. Ultrasonography also informs ovarian position to help guide incision location. Too large (mature) a stingray may have a large ovary with several cohorts of developing follicles making extraction more difficult and increasing the chance of hemorrhage during removal. Based on the authors’ experience, too small (immature) a stingray may increase the chance of leaving tissue that can become a functional ovary. Ultimately, the ideal stingray size was approximately 60–70 cm DW, at the onset of maturity, when ovarian margins are visually distinct from the epigonal and ovarian follicle development is minimal with the largest follicles 1 cm or less in diameter.

There were two mortalities among the first series of ovariectomies, both due to coelomitis. Both the procedure and equipment were refined with each procedure, and no further mortalities directly attributed to the surgery have occurred. During one of the initial procedures, an anesthetic hose popped out of the stingray’s spiracle and injected seawater into the surgery site. To prevent this problem during future procedures, anesthetic seawater pressure was decreased and a pre-moistened, rolled cotton towel was placed across the ray’s body just caudal to the spiracles. The towel should be pre-moistened to avoid stripping the stingray’s epithelial mucus and rolling the towel creates a protective barrier to block water reflux from the spiracle. Despite antibiotic therapy this animal died due to coelomitis. Southern stingrays did not appear to be adversely affected by the procedure and returned to normal swimming and eating behaviors the same day as the surgery.

Ovariectomy of southern stingrays is feasible within the realm of any experienced veterinary surgeon. Surgical time should be 45 min or less and with experience the procedure time should decrease to 30 min or less. Other institutions have used this procedure and successfully ovariectomized southern stingrays under their care to limit future reproduction (Dr. Shane Boylan, personal communication, 31 February 2024). Hysterectomy of the functional uterus and/or vestigial uterus was not considered for any ray because the primary purpose was to prevent reproduction which is accomplished via ovariectomy. Further, pathologies of the uterus related to reproductive disease are secondary to ovarian pathologies (5) and have not been observed for ovariectomized southern stingrays.

This surgical procedure can be extended for use in other ray species increasing the utility and impact of the procedure (7). It also could be used as a treatment to be employed successfully in older rays of various species with ovarian disease (Dr. Natalie Mylniczenko, personal communication, 19 March 2024). However, despite all rays being viviparous and histotrophic, their reproductive anatomy cannot be generalized. Rays have species-specific combinations of bilateral and unilateral ovarian and uterine functionality. In addition, ovarian location, attachment to peripheral organs and differences in vascular supply to the organ itself require confirmation before attempting ovariectomy. Further complicating the species-specific anatomy is potential ovarian plasticity, some ray species have asymmetric or even vestigial ovaries that may become functional if the dominant ovary is removed (not observed for southern stingrays) (7). Bilateral ovariectomy would require epigonal organ resection for at least one of the two paired myelopoietic organs making the procedure considerably more difficult to perform. Ovariectomy of southern stingrays is a safe and reliable method and management tool for husbandry professionals to control reproduction among their population.

## Data availability statement

The original contributions presented in the study are included in the article/supplementary material, further inquiries can be directed to the corresponding author.

## Ethics statement

The animal studies were approved by Ripley’s Aquariums Research Review Committee. The studies were conducted in accordance with the local legislation and institutional requirements.

## Author contributions

RG: Writing – original draft, Methodology, Conceptualization. CB: Writing – review & editing, Methodology. KB: Writing – review & editing, Methodology. JS: Writing – review & editing, Methodology. SW: Writing – review & editing, Data curation. TH: Writing – review & editing, Supervision. JW: Writing – original draft, Conceptualization.
